# Supplementary material for: Genomic surveillance of antimicrobial resistance shows cattle and poultry are a moderate source of multi-drug resistant non-typhoidal Salmonella in Mexico
Source: PLoS One. 2021 May 5;16(5):e0243681. doi: 10.1371/journal.pone.0243681 (PMC8099073; doi:10.1371/journal.pone.0243681)
Supplement: S2 Fig — (PDF) [file pone.0243681.s005.pdf]

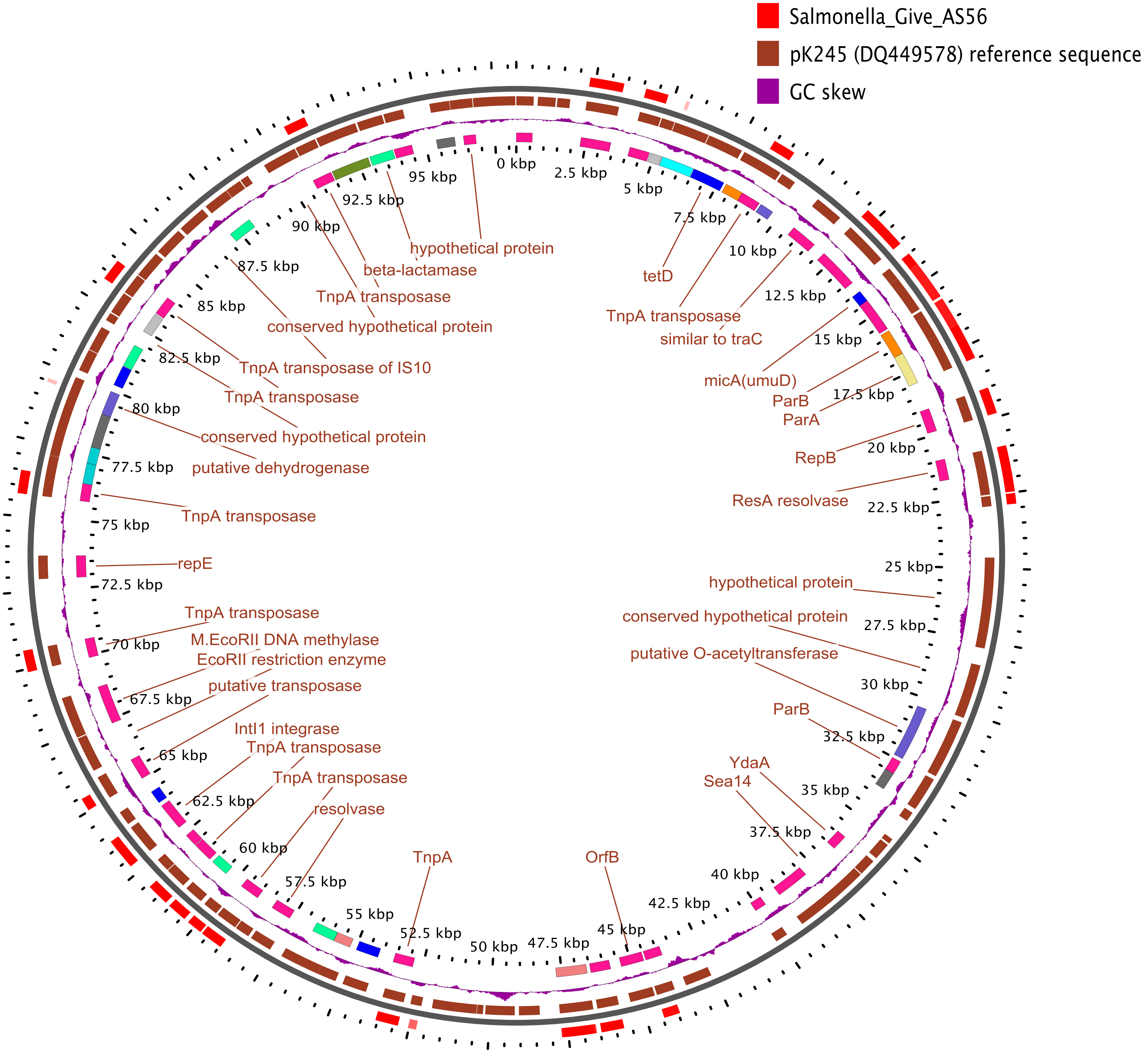

Figure S2. BLAST atlas of plasmid pK245 of one experimental serovar Give isolate. The black slot corresponds to the backbone and the inner ring to the reference plasmid sequence and genes. Map generated with GView, version 1.7 through the tblastx program, with e-value=0.001, alignment cutoff=50, identity cutoff=70, and no filtering of low complexity sequences. Refer to Table S1 for isolate accession and metadata.
