# Supplementary material for: Genomic surveillance of antimicrobial resistance shows cattle and poultry are a moderate source of multi-drug resistant non-typhoidal Salmonella in Mexico
Source: PLoS One. 2021 May 5;16(5):e0243681. doi: 10.1371/journal.pone.0243681 (PMC8099073; doi:10.1371/journal.pone.0243681)
Supplement: Attachment — Submitted filename: Response to reviewers R1.docx [file pone.0243681.s008.docx]

PONE-D-20-36934

Genomic surveillance of antimicrobial resistance shows cattle are a moderate source of multi-drug resistant non-typhoidal Salmonella in Mexico

PLOS ONE

The authors thank the reviewer and the academic editor for their valuable comments and suggestions that helped improve our manuscript. Below we list each comment raised during the reviewing process, followed by the authors’ responses**.**

**Academic Editor Comments (AEC)**

**AEC1.** Please ensure that your manuscript meets PLOS ONE's style requirements, including those for file naming. The PLOS ONE style templates can be found at

https://journals.plos.org/plosone/s/file?id=wjVg/PLOSOne_formatting_sample_main_body.pdf and

https://journals.plos.org/plosone/s/file?id=ba62/PLOSOne_formatting_sample_title_authors_affiliations.pdf

**Authors’ Response (AR)1.** We carefully reviewed the style requirements to make sure the manuscript meets them. Please, refer to the revised manuscript.

**AEC2**. We note that you are reporting an analysis of a microarray, next-generation sequencing, or deep sequencing data set. PLOS requires that authors comply with field-specific standards for preparation, recording, and deposition of data in repositories appropriate to their field. Please upload these data to a stable, public repository (such as ArrayExpress, Gene Expression Omnibus (GEO), DNA Data Bank of Japan (DDBJ), NCBI GenBank, NCBI Sequence Read Archive, or EMBL Nucleotide Sequence Database (ENA)). In your revised cover letter, please provide the relevant accession numbers that may be used to access these data. For a full list of recommended repositories, see http://journals.plos.org/plosone/s/data-availability#loc-omics or http://journals.plos.org/plosone/s/data-availability#loc-sequencing

**AR2.** The manuscript complies with this requirement. Please, refer to L121-131 (methods section) of the original manuscript. We declared raw sequences are publicly available at NCBI and provided the accession numbers and metadata in supplementary S1 Table. We also included the doi number for the two laboratory procedures that are citable and were uploaded at protocols.io.

**AEC3**. The reviewers have pointed out number of gaps in the manuscript. Clarifications and explanations are needed in all sections including methodology, results, discussion and data presentations in Tables and Figures. Please revise the manuscript considering all reviewer comments point by point.

**AR3.** The authors appreciate the time invested by the reviewers to make a very detailed and thorough revision of our manuscript. Comments will be taken into consideration to improve the paper and will be responded point by point.

**Reviewer Comments (RC)**

***RC1****. In this manuscript, the authors presented their phenotypic and genotypic AMR findings in NTS isolates derived from bovine lymph nodes and ground beef products in Mexico. They further compare their genotypic AMR findings with publicly available NTS genomes originated from Mexico. Overall, it is an interesting and valuable study that demonstrates the genomic similarities regarding the AMR profile between humans and bovine origin isolates in Mexico. However, I would suggest a major revision before publication. Please find more detailed comments below.*

**AR1**. We appreciate these comments and will address every observation to improve our manuscript.

***RC2****. Overall: First of all, authors were able to find 2400 publicly available Salmonella isolates at NCBI which 1,714 were from a known source, thus included in the study. Authors should include other sources that have the major number of isolates clustered within (for example avian source represented with BioProject PRJNA480281) to avoid selection bias while measuring “ source-related association with AMR Salmonella”. Please also help me to understand the “unknown source” criteria that yield exclusion of the isolates from this study. Please kindly provide your search criteria for the isolates. In addition, there are several major sources (e.g., papaya, pepper, river, canal) that were classified under vegetables or the aquatic environment in this study. Authors should count for any major source separately in the analyses to increase resolution. Please also consider adding the “unknown” source related data as “other sources”.*

**AR2.** We only used two selection criteria: location (isolates from Mexico), and isolation source (the record should have a declared, unambiguous isolation source). Isolates of “unknown source” are those for which the isolation source was missing or ambiguous (i. e. sponge, swab, meat, product, etc.) in the record. We were interested in analyzing the AMR genomic profile of bovine isolates in the context of the bigger *Salmonella* population circulating in other sources. Our aim was to avoid making reports that are too general in nature. Instead, we attempted to be as specific as possible, to be able to answer specific relevant questions, such as the following:

a) Are cattle a relevant source of AMR-*Salmonella*? Are they as relevant as other food-related sources and clinical cases?
b) What are the dominant genetic determinants associated with resistance to the studied antimicrobial classes?
c) How disseminated are these AMR genes within and across sources? How the situation compares with that in other countries/regions?
d) What factors are likely favoring AMR spread? What measures could be implemented to contain it?

The authors recognize committing several mistakes while conforming groups of isolates with the Excel file downloaded from NCBI. We overlooked the lack of uniformity in the records and relied on the “Filter” option of Excel to identify relevant groups. During this process, we missed all avian isolates, as well as some isolates from mostly every other source. We thank you for the detailed revision of the manuscript. In the updated version, we double checked the whole database to make sure there are no mistakes. Moreover, we agree to include all isolates from Mexico and thus, we created a new category named “Other sources, n=249”. This group includes isolates from sources with very few records, as well as those with ambiguous or unreported isolation source. Overall, the whole database includes 2,405 records now.

Regarding the comment on the need to break down categories into some major sources (i. e. papaya, pepper, river, etc.), we did the analysis separately before submitting the manuscript. While doing so, we observed there were minor differences within the vegetable and aquatic environment groups. So, we decided to analyze these categories as a whole for we did not see there was room for any resolution improvement. However, we decided to accept this comment and make the following adjustments:

The number of categories increased from 5 to 10:

1. Human, n=37

2. Bovine, n=179

3. Seafood, n=131. This category was not split since there was no variation in the AMR profile of isolates, regardless of the isolation source (fish, shrimp, others).

In the “vegetables” category, isolates from “papaya” were the only ones that differed from those from other vegetable sources in their AMR profile, although it happened only for aminoglycoside, quinolone and fosfomycin resistance genes. However, considering the number of “pepper” isolates was big, we split this category into three groups:

4. Papaya, n=279

5. Pepper, n=216

6. Other vegetables, n=568

The “aquatic environments” category was split into two groups, based on differences between surface waters and water from irrigation canals and other sources:

7. Surface water (rivers, ponds, lakes, dams), n=307

8. Other water sources (irrigation canals and other sources), n=246

Finally, two new categories were added:

9. Avian, n=193

10. Other sources, n=249

The methods section was updated to reflect these changes.

***RC3.*** *Authors should be able to report MLSTs of their isolates since they have already had WGS data. I believe this is highly important since AMR profiles and sub-serotypes are highly associated.*

**AR3.** Information on MLSTs is published in a previous paper on the epidemiology of *Salmonella* associated with bovine lymph nodes and ground beef [1]. This paper is cited in the introduction (L78-80). However, we agree to provide information on the STs of our experimental isolates in the subsection “*Salmonella* isolates” of the methodology, as well as to include STs in the discussion.

***RC4****.Another important aspect is the specific source of the 77 isolates should be provided along with the date of collection. These features need to be considered while driving any conclusion related to AMR profile and “source”. As a reviewer, I would like to see if the major group of Typhimurium isolates collected on 9/18/18 that show highly similar genotypic and phenotypic AMR profiles were all from the same batch of samples, city, or producer. Please clarify also in the manuscript and provide data.*

**AR4**. In connection with the previous comment, we included detailed information on the origin of isolates. Isolates from this project originated from the same company that integrates feedlot and slaughterhouse operations in the state of Veracruz. They sell carcasses in a wholesale store in Mexico City. All the samples originated from crossbred *Bos indicus* young bulls 24-36 months old. All the animals were harvested in the same slaughterhouse, while samples were collected at the store. Thus, all isolates originated from the same place, not only those of Typhimurium serovar. But they were collected across a 2-year period and all the isolates originated from a different sample. We agreed to include this information in the methods and discussion sections, for the sake of clarity. Please, refer to the updated manuscript to see the changes.

***RC5****. Abstract: Authors did not talk about 3GC in the abstract. Please mention the function/importance of ramR gene for MDR. The authors did not discuss their findings for high levels of amoxicillin-clavulanic acid-resistant isolates in the absence of AMR genes related to this phenotype in the manuscript.*

**AR5**. Since resistance to 3GC and carbapenem was low, we did not mention them in the abstract in order to meet the maximum allowed length, while including the most relevant information. For the same reason, we did not expand on the role of *ramR* gene in MDR phenotypes, as well as other findings. Nevertheless, the abstract was updated to include 3GC and carbapenems results.

Regarding the role of *ramR* gene mutations on MDR phenotypes, we did mention it in the manuscript (L444-446) and cited the paper that has a thorough explanation of the topic. However, we agree to broaden the discussion on *ramR* mutations, including their role in resistance to betalactams in isolates lacking ESBLs, as pointed out by the reviewer

***RC6.*** *Lines 100-102 Authors should explain why they choose these individual lists of antibiotics while there are many other antibiotics/classes listed in WHO. AMR in Salmonella is especially important when resistance emerges for the antibiotics that are used to treat Salmonella infections in humans. Macrolides (e.g., azithromycin) are one of the important antibiotics to treat Salmonella infections and this antibiotic was not included in this study.* *How would the authors explain their selection of antibiotics is unclear in the manuscript? Please also provide the class information of selected antibiotics either in the manuscript or table/figures.*

**AR6**. We included antibiotics that are in the WHO list of critically important and highly important antibiotics, according to WHO’s prioritization criteria [2]:

P1) High absolute number of people affected by diseases for which the antimicrobial is the sole or one of few therapies available.

P2) High frequency of use in human medicine.

P3) Evidence of transmission of resistant bacteria or AMR genes from non-human sources.

Thus, we picked some antimicrobials that are considered highest priority (i. e. aminoglycosides, carbapenems, quinolones), as well as those that are approved in Mexico for the treatment of both humans and food-producing animals (i. e. quinolones, 3GC, folate pathway inhibitors, etc.). Concerning azithromycin, it is only approved to treat cats and dogs in Mexico [3]. Moreover, according to a recent review covering the 2000-2017 period, azithromycin-resistant *Salmonella* has not been isolated from foods (including meats) in Mexico [4]. So, we decided to leave macrolides out of the AST panel. However, we know azithromycin resistant *Salmonella* is considered a public health risk in some countries, although it involves mainly typhoidal strains [5]. Hence, we limited macrolide analysis to comparative genomics, which confirmed there is a very low rate (<1%) of azithromycin resistant genotypes (*mphAB* and *lnuF* genes) among the public *Salmonella* isolates from Mexico studied here (n=2,405). This information was included in methods and results, while the discussion was broadened to include analysis of results on macrolide resistant genotypes. We also acknowledged this is a limitation of our study, considering the widespread distribution of *acrB* mutations, which have been reported to confer macrolide resistance in typhoidal strains [6] (refer to the “Discussion” section of the updated manuscript). Finally, the antibiotic class is not missing. We did provide that information in Fig. 1 label (L199-202 of the original manuscript). However, this figure was updated. We included all the details required for a proper interpretation of results, without having to consult the text.

***RC7.*** *Line 133- Please describe which parameter was used to detect the “poor quality reads used for trimming criteria*

**AR7**. We used the FastQC program, as declared in L132. This program analyzes raw read files and reports the quality score (Q score) across all bases, among other attributes. The Q scores report the probability of error in base calling ($P={10}^{{-Q}/{10}})$. So, reads that are going to be used for genome assembly should have a Q score of 30, ideally. In that way, we have a 99.999% confidence that the base called in that position is right. Below we provide an example of this report:

As you see, there is drop in the quality of base calling by the end of the insert (around position 155 and onwards). This is typical of raw reads obtained with Illumina technology, which uses the “sequencing by synthesis” approach. This requires adding adaptors to DNA fragments. So, that they can attach to the flow cell for DNA amplification and cluster formation. So, the adapters shall be removed before genome assembly. At the beginning, the quality of base calling is very good, as there is plenty of reagents and thus, every cluster flashes simultaneously when a new base is added. However, as reagents are consumed, some DNA fragments in the same cluster flash before others (pre-phasing) and/or some others flash later (phasing). This causes a fall in the quality of base calling, since the equipment cannot easily identify to which base the flash corresponds. Hence, researchers should make sure low-quality reads are removed before conducting genome assembly.

We also want to clarify that the minimum quality criterium was mentioned in the manuscript (L134, Q≥30). We did not provide a thorough explanation since this is a standardized procedure that it is not normally explained in papers. Please, refer to a recent publication in Plos One where the authors do not even mention quality assessment of raw reads [7]. Instead, they just refer the use of Trimmomatic to remove low quality reads. So, we do not believe expanding on this topic is necessary.

***RC8.*** *Line 136- Please provide the version of the Spade tool. Did the authors conduct a quality check for their assemblies?*

**AR8**. We included the Spades version in the methods section of the revised manuscript. The genome assembly service of PATRIC includes a QUAST quality report. This was also included in the methodology. We used to report that information in our papers, but it does not seem to be required by journals since anyone can download raw reads and reproduce the analysis. The Plos One paper we cited before [7] does not include this information either. However, we do have the relevant quality attributes of genome assembly (# of contigs, assembly length, GC, N50, L50) which were included in supplementary S1 file.

***RC9.*** *Line 147- Please refer to the human isolates as “human clinical cases” in related figures, tables, or manuscript as referred to in the line.*

**AR9**. After adjusting the groups of isolates, we decided to name this group just as “human isolates”. This new name is easier to refer in the text and similar to other sources (i. e. avian, bovine, etc.).

***RC10.*** *Line-149- Please provide the search term used to find these isolates and the criteria used. So, the readers can reach the same data analyzed in this study.*

**AR10**. Accepted.

***RC11.*** *Line 151- Please provide if the AMRFinderPlus database and program that was mentioned is the same one that is used in this analysis. Please provide the information of the data analyzed for this study that was scanned using the same database and version of the AMRFinderPlus. Since these results are prone to change as the AMR database and software are updated, authors must confirm that they compare the outcomes of WGS data using the same tools for all isolates included in the manuscript.*

**AR11**. Noted. The NCBI Pathogen Detection site is connected to the National Database of Antibiotic Resistant Organisms (NDARO) and to the FDA’s Global Resistome Data-Resistome Tracker Tool (https://www.fda.gov/animal-veterinary/national-antimicrobial-resistance-monitoring-system/global-resistome-data). Hence, the results they publish on AMR genotypes are kept and updated by the NCBI team, as required. In spite of this, we checked to make sure changes to AMRFinderPlus program and databases did not impact results for AMR genes. The program has been improved to include prediction of point mutations, as well as biocide, stress, heavy metal resistance and virulence genes. Please, refer to the github AMRFinderPlus releases page (<https://github.com/ncbi/amr/releases>) for a thorough report of the changes made across versions. In addition, we tested whether results would change across versions. For instance, we got the same results for our 77 isolates by running AMRFinderPlus locally (version 3.8.4) as compared to those reported by NCBI for the same isolates (they used version 3.6.7). Likewise, we got the same results reported by NCBI for 32 isolates (analyzed with the earliest version 3.2.3 among isolates from Mexico) while running these genomes with version 3.8.4 locally. Taking this into consideration, we decided to add a brief explanation on AMR genotypes published by NCBI and the analysis we conducted to make sure AMR gene profiling is not affected by the program version or database, among this group of isolates.

***RC12.*** *Line 152- Please provide the methodology for serotyping*

**AR12**. This information was already published in our previous research that we cited on the methods section [1]. We performed *in silico* analysis to predict serovar by running raw reads on the SeqSero software, version 1.2 [8]. However, for the sake of clarity, we added information on how serotyping was conducted in the methods section. Please, refer to the section “*Salmonella* isolates”.

***RC13.*** *Line 166- Please explain how the AMR profiles of each plasmid were determined.*

**AR13***.* Plasmid profiling is different from AMR profiling. As mentioned in the original manuscript (L167-169), we used PlasmidFinder program, version 2.1, to predict the plasmid profile of each isolate. If the program detected plasmid replicons in a genome, then we collected the reference sequence of each predicted plasmid from NCBI, which contains all the coding sequences of the plasmid, including those related to AMR. So, there is no need to determine the plasmid’s AMR profiles. Just by looking at their reference sequences we can determine if they harbor AMR genes. The objective of plasmid profiling is to check if the isolates are likely carrying plasmids.

***RC14.*** *Line 171- Why the authors select the %70 thresholds?*

**AR14.** The 70% cutoff is arbitrary. It aims to establish that the majority of a reference plasmid sequence must be present in a draft genome to propose that the genome actually carries that plasmid. WGS data includes both chromosomal and plasmid DNA (if the strain carries a plasmid). Although cumbersome, it is possible to predict the presence of plasmids in a draft genome by combining different lines of evidence:

1) **Detection of plasmid replicons in the genomes**

2) **Tiling contigs against reference plasmids**. If we identified consecutive genes that were in the same genomic context in contigs and plasmids, and the majority of the reference plasmid is covered in the genome, these were proposed to be plasmids in that genome.

3) **Average depth of coverage**. Normally, plasmid-associated contigs have the same or very similar read depths between them, and different from that of chromosomal contigs. There are few exceptions, such as multi-copy ribosomal genes, as well as big plasmids, which may have similar read depths as compared to chromosomal DNA. However, in general, this approach works fairy well and checking read depth is only required when contigs do not tile well against reference plasmids. We started to use this method based on a paper published before in Plos One [9]. It has been accepted in papers published by our research group in Scientific Reports [10] and Journal of Microbiology [11]. The manuscript provides accessions of both our draft genomes and plasmids. So, that anyone can reproduce the analysis. For the sake of clarity, we described the methodology in full and cited previous papers using the same approach.

***RC15.*** *Line 175-180 These lines are misleading. There is no phenotype data included in the analysis of publicly available data. Please modify it accordingly.*

**AR15.** Accepted. The text was modified to make clear which analyses were conducted with our isolates and which ones with public isolates.

***RC16.*** *Line 181- Even though the statistical language is sometimes used loosely in this regard, the authors performed analyses of association - not correlation - between phenotype and genotype and they will probably wish to change the wording to reflect that important difference.*

**AR16***.* We calculated the Pearson correlation coefficient between the % of phenotypically non-susceptible isolates and the % of genotypically non-susceptible isolates for each of the tested antibiotics (Fig. 2). Based on the previous comment, we modified the wording of this whole section for the sake of clarity.

***RC17.*** *Line 512-513 I would suggest authors review their statement related to WGS is being a better tool to monitor aminoglycoside resistance as compared to AST. The area of aminoglycoside resistance is difficult and evolving and interpretations are difficult and changing. Nonetheless, there is apparent cryptic genes aac(6’) and other factors such as breakpoints set for resistance for which phenotype and genotype seem disconnected. This disagreement does not infer that WGS is better, assuming that in at least some cases the naming conventions for the genes themselves might be based on flawed original experimentation, or else based originally in another genus or species.*

**AR17**. Accepted. That statement was removed from the text.

***RC18.*** *Line 520 Please provide how many isolates had ramR mutations and did not harbor AMR genes but were found phenotypically resistant to chloramphenicol to support this statement.*

**AR18**. This statement is based on the strong association between chloramphenicol resistance and *ramR* mutations that we reported in the “Results” section of the original manuscript (L333-335). However, we agreed to provide detailed information on the predicted mutations for each isolate, as recommended by the reviewer (see the updated Fig 1).

***RC19.*** *Line 538-577 Authors may revise their justification related to the MDR presence in lymph node and ground beef as fecal origin Salmonella may travel from intestine to LN via payer’s patches, and transmission of environmental Salmonella may most likely occur via fly bites in the feedlots. Most importantly, lymph nodes are the most likely source of Salmonella found in ground beef products and contamination can occur via the fat trimming process.*

**AR19**. We agree lymph nodes (LN) contribution to *Salmonella* contamination in ground beef is well documented. This is not questioned in the manuscript. However, we did find a strong association between source of isolates and the proportion of MDR strains (L215-217), with a higher probability of finding MDR isolates in ground beef. Moreover, LN are not the sole source of *Salmonella* in ground beef, especially in developing countries, where the rate of carcass contamination (which is mainly of fecal origin) is higher as compared to that observed in industrialized nations. For instance, there are Mexican studies reporting a *Salmonella* prevalence of 6-18% in beef carcasses [12-14], while in ground beef the rates may go above 60-70% [4, 13, 15]. Thus, fecal contamination is likely contributing to *Salmonella* contamination in ground beef in countries with a similar situation. This is further supported by our previous findings [1], showing some *Salmonella* serovars (i. e. Typhimurium) were only detected in ground beef samples but not in LN from the same carcass, while some others were exclusively present in LN (i. e. Kentucky, Fresno, Muenster, Give) and not in ground beef obtained from the same carcass. Nonetheless, we revised the wording of this section to temper the statements and make sure they are just used to put the research into context without implying a challenge to the well-known contribution of LN to ground beef *Salmonella* contamination.

**RC20**. *Table 1 – Please explain “C”*

**AR20**. There is a superscript in the footnote of Table 1 indicating it refers to the antimicrobial’s disk concentration.

**RC21**. *Table 2- is confusing. Please consider reporting the point mutations and observed phenotype observed in 77 isolates that were not related to a defined AMR gene.*

**AR21**. Accepted. This table was removed, and its data were incorporated in Fig 1, as suggested by the reviewer in other comments.

**RC22.** *Table 3- how did the author screen the plasmidal content? Please include in the methodology*.

**AR22**. This information was included in the methodology (L166-173 of the original manuscript). We used PlasmidFinder program, version 2.1. Considering this comment, as well as RC14 above, we decided to expand the description of these methods in the updated manuscript.

**RC23.** *Figure 1- This table is hard to read. Authors may consider placing the class of phenotypic and genotypic resistance data next to each other for each class. I believe the qacEDelta1 is irrelevant with this research and is not shown on the AMRFinderPlus outcome of the isolates based on my research for given SAM IDs. Please help me to understand how this gene was found and why it was related to* this study. Please also add ramR column here since the relationship between the *mutation and phenotype has been analyzed and discussed.*

**AR23**. The table was updated considering this reviewer suggestions and we also included information on point mutations, as well as MDR phenotypes. Regarding the *qacEDelta1* gene, it can be predicted from assembled genomes, together with other biocide, stress, and heavy metal resistance genes, if the analysis is run with the --plus option. Please, refer to the github AMRFinderPlus repository for usage options (<https://github.com/ncbi/amr/wiki/Running-AMRFinderPlus#usage>). In a previous study [11], we observed there was association between the presence of *qacEDelta1* gene and MDR phenotypes. The stress induced by many factors, including biocides, is known to trigger AMR resistance in bacteria [16]. Moreover, some of these genes are frequently found in isolates carrying class-1 integrons, which are known to play a key role in bacterial AMR [37]. Thus, we aimed to assess if biocide resistance genes were disseminated among experimental isolates showing MDR phenotypes. So, we decided to run AMRFinderPlus with the --plus option, to check if there was association between the presence of this gene and the observed phenotypes. Nevertheless, considering this gene was present in just a few experimental and public isolates, we agree to remove it from the table.

**RC24***. Figure 2- Please provide the number of isolate information for each antibiotic. Even though the statistical language is sometimes used loosely in this regard, the authors performed analyses of association - not correlation - between phenotype and genotype and they will probably wish to change the wording to reflect that important difference.*

*Please consider using singular for sources. I would suggest revising the presentation of the source of isolates such as (e.g. human, bovine). Are these isolates have all genes listed in the conferring header (e.g., tetABCDGM) or at least one of them? Please clarify and add a footnote as needed.*

**AR24**. The label of Figure 2 mentions the total number of isolates (n=77, L222 of the original manuscript) and the figure reports the % of isolates with phenotypic and genotypic resistance to each tested antibiotic. So, the number of isolates can be calculated. Moreover, we are reporting the results of a Pearson correlation analysis, as mention before. This was clarified in the methods section. To facilitate the interpretation of these results, however, we modified Figure 2 and it is now reporting the number of non-susceptible isolates in each antibiotic, instead of the proportion. Moreover, we included the Pearson correlation coefficient and the corresponding P-value in the graph.

We understand the second part of this comment refers to Fig 3. Authors agreed to name sources in singular. Moreover, the source of every isolate was double checked to make sure the information is in accordance with the recorded isolation source at NCBI. For the heatmap, we grouped AMR alleles according to the resistance mechanism they encode. Regarding tetracycline resistance, we only detected AMR genes encoding efflux mechanisms (*tetABCDGM*). Thus, isolates were considered genotypically resistant if they carry at least one of these genes. We agree this needed clarification and thus, this figure was modified considering previous comments, as well as those stated here.

**RC25**. *S1 Table- please revise the number of sample size in the tab. I would suggest removing the collection date from Table 1 since it was not discussed in the manuscript. Please also provide the date of isolation and if possible, the information about the source of isolates (if they were collected from the same city/abattoir etc.) of your isolates in S1*.

**AR25.** The sample size in the tab is an editing mistake. Furthermore, in NCBI metadata, isolation date and collection date are the same. In fact, there is no “isolation date” field at the NCBI Pathogen Detection website. In connection with previous comments, the methods and discussion sections were modified to include information about the source of isolates. The influence of collection dates is discussed in our previous paper [1]. But it only refers to a seasonality in *Salmonella* prevalence, which is higher in warm climate as compared to winter. Therefore, we are now reporting the association between AMR profiles and season or year of collection. This information was not mentioned in the original manuscript. So, it was now incorporated in the results and discussion sections.

**RC26**. *I would also suggest providing the phenotypic data in S1 along with the ramR mutation*.

**AR26.** We do not accept this suggestion. Data on AMR phenotypes and mutations were incorporated in the main body of the manuscript. So, there is no need to duplicate that information. Thus, we decided to remove the AMR genotypes from S1 Table instead.

**RC27**. *There are duplicated gene names (.e.g., sul1), please correct.*

**AR27**. We would like to clarify that the duplicated *sul1* gene in some isolates is not a mistake. This only happened in isolates of serovar Typhimurium carrying the resistance island SGI1. Please, refer to Figure 4 where you can check that this gene is actually duplicated in SGI1. This can also be checked by accessing the SGI1 reference sequence (AF261825.2), which is provided in Figure 4 as well.

However, AMR genotypes were removed from S1 Table to avoid duplications with figures from the main body of the manuscript.

**RC28**. *Please include the AMR related genes and mutations and isolate names as shown in Table 1 as they are not matching with supplemental material (the last column in Table 1 is missing*.

**AR28**. We understand the reviewer refers to Figure 1 when citing Table 1. As mentioned before, AMR genotypes were removed from S1 Table to avoid duplicating information with the main body of the manuscript. The purpose of S1 Table is to comply with data availability requirements. All tables and figures were revised to make sure isolate names are used uniformly across the manuscript.

**RC29**. *I would also suggest using the same isolate names consistent in the manuscript and related data.*

**AR29.** Accepted. The tables were updated according to this comment.

**RC30**. *Many cells in the spreadsheet are missing, please revise.*

**AR30**. This table was prepared by downloading our own isolates from the NCBI Pathogen Detection website. While submitting raw reads to NCBI, some laboratories fail to indicate the serovar. In other cases, NCBI do not assemble some isolates. This is the reason why there are blank cells, corresponding to records with no serovar recorded in the metadata, as well as empty Assembly and WGS accessions, for those isolates that have not been assembled by NCBI so far. Again, since the purpose of S1 Table is to comply with data availability, we decided to provide the information as it appears at the public repository. However, we updated this table and left only relevant information that allows readers to reach the data.

**RC31**. *There are grammatical issues that need to be corrected. Authors need to revise AMR gene names and make sure they are presented correctly and consistently in both the body manuscript and related data.*

**AR31**. The manuscript was carefully revised to correct grammatical mistakes. We used gene names as reported by AMRFinderPlus and published at the NCBI pathogen detection website. Both the manuscript and supplementary files were checked to make sure they are correctly and consistently used.

**RC32.** *S2 Table- Please correct the name on the tab. There are “environment or environmental sample” related isolates classified as the aquatic environment. Please revise to verify these isolates are actually from an aquatic source.*

**AR32**. Noted. The name on the tab was left by mistake. It was removed. There are indeed some isolates recorded as “environment or environmental sample” that were collected from water samples, as indicated in their Biosample records. Nevertheless, according to previous comments on this issue, all source categories were revised, and corrections were made in the updated version of S2 Table.

**RC33**. *S3 Table- Please revise the AMR genotype column as there are duplicates, and information not relevant to AMR genes*

AR33. This table is reporting information as it appears at the NCBI Pathogen Detection website. When there are duplicated genes, this should be related to the presence of integrons containing duplicated AMR gene cassettes in that isolate. As occurs in the SGI1 integron commented before (see AR27), this is not a mistake.

**RC34**. *Reference: Overall- References are not standardized, please correct the inconsistency observed with the lower- and upper-case use. Please revise all the links provided (e;g., URL of Ref 44 is not working) and provide accession dates for each URL. Please also italicize the spp names as needed.*

**AR34**. References were generated automatically by using the last version of Plos reference style in EndNote X9.3.3, as recommended by Plos One. We also checked recent papers published at Plos One (i.e. DOI: <https://doi.org/10.1371/journal.pone.0244057>) and they also lack uniformity in the lower- and upper-case use. Probably, this inconsistency is caused by the way EndNote or similar programs capture the title of each publication. We thought this was acceptable considering there are published papers with these inconsistencies and some others. For instance, in the above referred paper, journal names are not abbreviated, which is supposed to be a Plos One requirement. Instead, they use a “Sentence case” style, with only the first word of the journal name capitalized and the text is italicized. In any case, we carefully reviewed this section to remove any mistake or inconsistency pointed by the reviewer. The URL of Ref 44 was recently updated by the government. We checked again every URL provided to make sure they all work and added the accession dates.

1.	Palós Gutiérrez T, Rubio Lozano MS, Delgado Suárez EJ, Rosi Guzmán N, Soberanis Ramos O, Hernández Pérez CF, et al. Lymph nodes and ground beef as public health importance reservoirs of *Salmonella* spp. Revista Mexicana de Ciencias Pecuarias. 2020;11(3):795-810. doi: 10.22319/rmcp.v11i3.5516.

2.	WHO. Critically important antimicrobials for human medicine. 6th Revision 2018: World Health Organization; 2019. Available from: <https://www.who.int/foodsafety/publications/antimicrobials-sixth/en/>.

3.	SADER. Productos químico-farmacéuticos vigentes: Secretaría de Agricultura y Desarrollo Rural, Gobierno de México; 2020 [cited 2020 September 20, 2020]. Available from: <https://www.gob.mx/cms/uploads/attachment/file/512374/PRODUCTOS_VIGENTES_QF_2019.pdf>.

4.	Godinez-Oviedo A, Tamplin ML, Bowman JP, Hernandez-Iturriaga M. Salmonella enterica in Mexico 2000-2017: Epidemiology, Antimicrobial Resistance, and Prevalence in Food. Foodborne Pathog Dis. 2020;17(2):98-118. Epub 2019/10/28. doi: 10.1089/fpd.2019.2627. PubMed PMID: 31647328.

5.	McDermott PF, Zhao S, Tate H. Antimicrobial Resistance in Nontyphoidal Salmonella. Microbiol Spectr. 2018;6(4). Epub 2018/07/22. doi: 10.1128/microbiolspec.ARBA-0014-2017. PubMed PMID: 30027887.

6.	Hooda Y, Sajib MSI, Rahman H, Luby SP, Bondy-Denomy J, Santosham M, et al. Molecular mechanism of azithromycin resistance among typhoidal Salmonella strains in Bangladesh identified through passive pediatric surveillance. PLoS Negl Trop Dis. 2019;13(11):e0007868. Epub 2019/11/16. doi: 10.1371/journal.pntd.0007868. PubMed PMID: 31730615; PubMed Central PMCID: PMCPMC6881056.

7.	van den Berg RR, Dissel S, Rapallini MLBA, van der Weijden CC, Wit B, Heymans R. Characterization and whole genome sequencing of closely related multidrug-resistant Salmonella enterica serovar Heidelberg isolates from imported poultry meat in the Netherlands. PLOS ONE. 2019;14(7):e0219795. doi: 10.1371/journal.pone.0219795.

8.	Zhang S, Yin Y, Jones MB, Zhang Z, Deatherage Kaiser BL, Dinsmore BA, et al. Salmonella serotype determination utilizing high-throughput genome sequencing data. J Clin Microbiol. 2015;53(5):1685-92. doi: 10.1128/JCM.00323-15. PubMed PMID: 25762776; PubMed Central PMCID: PMCPMC4400759.

9.	Dhanani AS, Block G, Dewar K, Forgetta V, Topp E, Beiko RG, et al. Genomic Comparison of Non-Typhoidal Salmonella enterica Serovars Typhimurium, Enteritidis, Heidelberg, Hadar and Kentucky Isolates from Broiler Chickens. PLoS One. 2015;10(6):e0128773. doi: 10.1371/journal.pone.0128773. PubMed PMID: 26083489; PubMed Central PMCID: PMCPMC4470630.

10.	Delgado-Suárez EJ, Selem-Mojica N, Ortiz-López R, Gebreyes WA, Allard MW, Barona-Gómez F, et al. Whole genome sequencing reveals widespread distribution of typhoidal toxin genes and VirB/D4 plasmids in bovine-associated nontyphoidal Salmonella. Sci Rep. 2018;8(1):9864. doi: 10.1038/s41598-018-28169-4.

11.	Delgado Suárez EJ, Ortíz López R, Gebreyes WA, Allard MW, Barona-Gomez F, Salud Rubio MS. Genomic surveillance links livestock production with the emergence and spread of multi-drug resistant non-typhoidal Salmonella in Mexico. J Microbiol. 2019;57(4). doi: DOI 10.1007/s12275-019-8421-3.

12.	Narvaez-Bravo C, Miller MF, Jackson T, Jackson S, Rodas-Gonzalez A, Pond K, et al. Salmonella and Escherichia coli O157:H7 Prevalence in Cattle and on Carcasses in a Vertically Integrated Feedlot and Harvest Plant in Mexico. J Food Prot. 2013;76(5):786-95.

13.	Martinez-Chavez L, Cabrera-Diaz E, Perez-Montano JA, Garay-Martinez LE, Varela-Hernandez JJ, Castillo A, et al. Quantitative distribution of Salmonella spp. and Escherichia coli on beef carcasses and raw beef at retail establishments. Int J Food Microbiol. 2015;210:149-55. Epub 2015/07/01. doi: 10.1016/j.ijfoodmicro.2015.06.016. PubMed PMID: 26125489.

14.	Perez-Montaño JA, González-Aguilar D, Barba J, Pacheco-Gallardo C, Campos-Bravo CA, García S, et al. Frequency and Antimicrobial Resistance of Salmonella Serotypes on Beef Carcasses at Small Abattoirs in Jalisco State, Mexico. J Food Prot. 2012;75(5):867-73. doi: 10.4315/0362-028X.JFP-11-423.

15.	Cabrera-Diaz E, Barbosa-Cardenas CM, Perez-Montano JA, Gonzalez-Aguilar D, Pacheco-Gallardo C, Barba J. Occurrence, serotype diversity, and antimicrobial resistance of salmonella in ground beef at retail stores in Jalisco state, Mexico. J Food Prot. 2013;76(12):2004-10. doi: 10.4315/0362-028X.JFP-13-109. PubMed PMID: 24290673.

16.	Poole K. Bacterial stress responses as determinants of antimicrobial resistance. J Antimicrob Chemother. 2012;67(9):2069-89. doi: 10.1093/jac/dks196. PubMed PMID: 22618862.
