# Supplementary material for: Genomic surveillance of antimicrobial resistance shows cattle and poultry are a moderate source of multi-drug resistant non-typhoidal Salmonella in Mexico
Source: PLoS One. 2021 May 5;16(5):e0243681. doi: 10.1371/journal.pone.0243681 (PMC8099073; doi:10.1371/journal.pone.0243681)
Supplement: Attachment — Submitted filename: Response to reviewers R2.docx [file pone.0243681.s009.docx]

PONE-D-20-36934R1

Genomic surveillance of antimicrobial resistance shows cattle are a moderate source of multi-drug resistant non-typhoidal Salmonella in Mexico

PLOS ONE

The authors thank the academic editor and the reviewer for their detailed review. Below we list each comment raised during the reviewing process, followed by the authors’ responses**.**

**Academic Editor Comments (AEC)**

**AEC1.** Journal Requirements

Please review your reference list to ensure that it is complete and correct. If you have cited papers that have been retracted, please include the rationale for doing so in the manuscript text, or remove these references and replace them with relevant current references. Any changes to the reference list should be mentioned in the rebuttal letter that accompanies your revised manuscript. If you need to cite a retracted article, indicate the article’s retracted status in the References list and also include a citation and full reference for the retraction notice.

**Authors’ Response (AR)1.** As far as we know, we did not cite retracted papers. However, we carefully reviewed each cited reference again and none have been retracted. We found five papers that have been corrected but just for editing mistakes that do not affect the citation made in our manuscript. We also reviewed the reference list to make sure it is complete and correct. Please, refer to the revised manuscript. Finally, we added two new references (#24 and 25 in the revised version):

24. Zhang S, den Bakker HC, Shaoting L, Chen J, Dinsmore BA, Lane C, et al. SeqSero2: rapid and improved *Salmonella* serotype determination using whole genome sequencing data. Appl Environ Microbiol. 2019;85:e01746-19. doi: <https://doi.org/10.1128/AEM.01746-19>. PMID: 31540993
25. Yoshida CE, Kruczkiewicz P, Laing CR, Lingohr EJ, Gannon VPJ, Nash JHE, et al. The *Salmonella* in silico typing resource (SISTR): an open web-accessible tool for rapidly typing and subtyping draft *Salmonella* genome assemblies. PLoS One. 2016;11(1):e0147101. doi: 10.1371/journal.pone.0147101. PMID: 26800248

These references were added in connection with RC6 and AR6.

**AEC2**. The reviewers have pointed out some mismatches between the statements and data including those in supplementary Table. Please correct these discrepancies and other points raised by the reviewers.

**AR2.** The authors appreciate the time invested by the reviewers to make a thorough revision of our manuscript. Comments will be taken into consideration to improve the paper and will be responded point by point.

**Reviewer Comments (RC)**

***RC1****. Fig 1. Some of the phenotypic resistance (e.g., STX) related cells were highlighted (filled), please be consistent. This is not matching with author’s statement for Fig 1. “ For AMR genotypes, cells filled with the corresponding antibiotic class color indicate the gene is present.” Authors may also consider rearranging the order of the isolates by serotype followed by day and sample type. So, the readers can associate the time of collection and sample type data along with the resistance profiles observed.*

**AR1**. Accepted. These editing mistakes were corrected in the revised version. Regarding the rearrangement of isolates, they were already ordered by serovar and collection date. It is not possible to include sample type as another ordering criterium (for the whole set) without changing the previous order by collection date. Hence, the rearrangement was conducted within each serovar and now the collection date is ordered within sample type.

***RC2****. Please correct “metadata” in “S3 File. NCBI accesions, metada and antimicrobial resistance genotypes of fully sequenced public Salmonella enterica ser. Typhimurium isolates from Mexico included in this study”. Also, please check other instances.*

**AR2.** Accepted. We corrected this editing mistake in S3 File and also reviewed all the supplementary files to make sure there were no overseen editing mistakes left.

***RC3.*** *There are few S. Typhi included in the supplemental data, since the manuscript is about NTS, please revise accordingly.*

**AR3.** We acknowledge typhoidal strains, as human-restricted pathogens, are not commonly transmitted through food. However, in developing countries (such as Mexico), there is a high number of street vendors with poor hygienic practices and practically no health control. Hence, it is not possible to discard that some typhoidal strains involved in clinical cases may be foodborne. Especially, considering the Mexican surveillance system does not have attribution data. This is the reason why we included typhoidal strains in the analysis. However, removing these strains from the analysis will not change results, while improving the clarity of the manuscript in regard to its scope. Hence, we accepted to remove typhoidal strains from the analysis and Fig 3 was updated accordingly.

***RC4****.* *S2 file. There are about 112 isolates that have missing AMR data, please either remove or justify adding these strains in the metadata.*

**AR4**. This is not a mistake. These isolates were not predicted to carry any AMR genes. That is why their AMR genotype cells were empty. As mentioned in the previous review round, this report matches the structure of metadata reported at the NCBI pathogen detection site. For the sake of clarity, we added the following note at the heading of this file: “**Metadata is reported as recorded at the NCBI pathogen detection website. Blank cells in any column indicate this information is not available at NCBI. Isolates with blank cells under the AMR genotypes column were not predicted to carry any AMR gene**”.

***RC5****. In the S2 file, there are 46 Typhimurium isolates from Mexico, authors include 38 of them in the S3 file. Did I miss anything? I am asking this question based on their statement: “Furthermore, considering the epidemiological importance of this serovar, we also analyzed the whole set of Typhimurium isolates from Mexico deposited at NCBI (n=38, refer to S3 Table for accession numbers and AMR genotypes of this group of isolates).”*

**AR5**. While recovering Typhimurium isolates from Mexico at the NCBI pathogen detection site, we used the plain serovar name “Typhimurium”. We did not include any variant, such as var. Copenhagen (there are four isolates of this variant in S2 File) or the monophasic 1,4,[5],12:i:-. One of our isolates is monophasic but is recorded as Typhimurium at NCBI. At the moment this isolate was submitted to NCBI by the Mexican Department of Agriculture, it was predicted as Typhimurium, based on *in silico* analysis of raw reads. As we will explain later in this document (see AR6), after assembling genomes and running serovar prediction analyses with SeqSero2 and SISTR programs, the serovar of this isolate was corrected to monophasic 1,4,[5],12:i:-. Hence, there are five isolates in S2 file that are Typhimurium variants and thus, they were not included in the Typhimurium set.

Following this comment, we carefully checked S2 File and there are 45 records (not 46) that contain Typhimurium in the serovar field. After removing the variants, there are 40 Typhimurium instead of 38. During the previous review round, we updated the whole dataset. As a result, the number of isolates identified increased in almost all categories. Particularly, there were two additional Typhimurium isolates of human origin that were added and we forgot to update S3 File accordingly. This is the reason why we have the mismatching. We thank you for the detailed revision. The methods section was updated to indicate we identified 40 Typhimurium isolates (instead of 38). Likewise, the results section and S3 file were adjusted to reflect these changes and eliminate the mismatching.

***RC6.*** *I am also having difficulty matching the selected genomes (n=77) from the previous study (ref #18), with the current study. Authors state: “In the present investigation, we conducted antibiotic susceptibility testing and WGS of 77 NTS isolates collected in the course of a previous research project involving bovine lymph nodes (n=800) and ground beef (n=745) across a two-year sampling period [18]” and also states “ We identified nine Salmonella serovars: Anatum (n=23), Reading (n=22), Fresno (n=4), Typhimurium (n=10), London (n=9), Kentucky (n=6), and Muenster, Give and monophasic Typhimurium 1,4,[5],12:i:- (one each)”. However, the previous paper referred by authors states “78 isolates obtained from the 1,545 samples analyzed in the two years” and in the same paper, there are Reading(n=23), Anatum (n= 23), Typhimurium (n= 11), London (n= 9), Muenster (n= 2), Kentucky (n= 5), Give (n=1), and Fresno (n=4) serotypes. There was also no monophasic serovar identified in the previous manuscript. In this current version – if authors claim they include isolates from previous study – they should clarify why there is a mismatch exist with the isolates corresponding.*

**AR6**. Our previous study (ref #18) was written and published when we only had the raw reads available. While using raw reads in SeqSero for serovar prediction, the monophasic Typhimurium isolate was predicted as Typhimurium. After assembling genomes, we repeated the analysis for all isolates both with SeqSero2 and SISTR programs (two references #24 and 25 were included to cite the source of both. programs). By doing so, we confirmed serovar prediction results, which were mostly consistent with results reported in ref#18, except for the monophasic Typhimurium and one isolate predicted as serovar Reading that we had to discard for having poor assembly quality and inconsistent results with *Salmonella* species (genome size >8 Mb and GC content of 46.5%). Due to the COVID-19 pandemia, this isolate has not been re-sequenced yet. We are currently waiting for re-sequencing results before filing an amendment request to the publisher of ref#18. In the meantime, however, we decided to exclude this isolate from the present manuscript. This clarification was included in the methods section.

***RC7.*** *I see in the S1 file, the strain UNAM2018123_Sa_AN13 was marked as monophasic, however, in the S2 file, this strain was recorded as Typhimurium. And finally, at the S3 file, while all Typhimurium isolates were included from “Mexico” based on the metadata (S2 file), this was excluded. I am really confused. If this strain was later identified (or corrected) as monophasic, how the authors make sure the Typhimurium strains in the S3 file are all monophasic? Please clarify this for me if there is a misunderstanding or if I am missing anything, otherwise, please provide your selection criteria and your confirmation method used for strains reported in the S3 file.*

**AR7**. We are sorry to have caused such a confusion. S2 file contains information as recorded at the NCBI pathogen detection site. When isolates included in this manuscript were uploaded to NCBI, the submitter (the Mexican Department of Agriculture) included the serovar results we had at that moment. Therefore, isolate UNAM2018123-Sa-AN13 was recorded as Typhimurium. However, while preparing the current manuscript, and redoing serovar prediction analyses with assembled genomes in two different programs, we confirmed this isolate is indeed monophasic Typhimurium. That is why S1 File reports the correct serovar for this isolate. S3 File only reports Typhimurium isolates (not variants) available at NCBI. We did not change this record in S2 file for it would not match the actual record at NCBI. However, we already asked the Mexican Department of Agriculture to file an amendment request to NCBI, so that this record is corrected. Therefore, we decided to correct this record in S2 File as well, to avoid confusing the readers. In the updated version of the manuscript, including supplementary information, isolate UNAM2018123-Sa-AN13 is reported as monophasic Typhimurium. Moreover, we added the following note at the heading of the bovine isolates section of S2 File: “**Isolate with Biosample accession SAMN12857424 (strain UNAM2018123_Sa_AN13) was recorded at NCBI as serovar Typhimurium at the moment its raw reads were submitted. However, further analyses with the assembled genome showed it is a monophasic Typhimurium variant (1,4,[5],12:i:-). This record should be corrected soon at the NCBI pathogen detection site**.”

***RC8.*** *Once again, please fill the blank cells in the metadata as either “not reported” or provide a footnote for those cells in all S-related files. It is important for readers to understand why the cells were left blank.*

**AR8**. As mentioned in AR4, we included a note below the heading of S2 file. We have also included a similar note below the heading of S3 files: “**Metadata is reported as recorded at the NCBI pathogen detection website. Blank cells in any column indicate this information is not available at NCBI**”. S1 file does not have any blank spaces.

***RC9.*** *Authors stated “ For instance, most serovar Typhimurium isolates (9/10) carried SGI1”. However, in Fig 4, all 10/10 isolates were showing SGI1. Please clarify this.*

**AR9**. According to Fig 4, only 9 isolates carried SGI1. Please, re-check. Fig 4

includes the 10 Typhimurium isolates as well as the monophasic variant. We included the variant in this analysis since it resisted several antimicrobials and we wanted to assess if it also carried SGI1. Please, notice there is one Typhimurium isolate (AK68) and the monophasic variant (AN13) lacking SGI1 (first two rings after the backbone). After these two, there are only 9 rings, corresponding to the remaining Typhimurium isolates.

***RC10.*** *Please consider to include the SGI1, AMR and phenotype info along with the ramR mutations in S1 file, it is very hard for the readers to match individual IDs and serotypes with corresponding data in the S1.*

**AR10**. We do not see the need to duplicate the information in the main body of the manuscript and supporting files. In the main body, Fig 1 reports AMR phenotypes and mutations, as suggested by the reviewer previously. Likewise, Fig 4 reports the isolates carrying SGI1. Both figures report isolate names and serovars, which are also included in S1 File. Hence, we do not see why it would be difficult for readers to match individual IDs and serovars with information reported in S1 file. As mentioned in the previous review round, S1 File is meant to comply with data availability and facilitate reproducibility.

***RC11.*** *S1.File genome size cells need correction.*

**AR11**. Our Excel program uses Spanish as the default number format (apostrophes to separate millions and commas to separate thousands). We corrected the file to comply with US format.

***RC12.*** *Please also revise the abbreviations used and use the original names at the first instances.*

**AR12**. Accepted. We carefully reviewed the manuscript to make sure original names are provided at the first instances. In some cases, such as in figures and tables headings, original names and abbreviations are repeated to facilitate the interpretation of figures and tables without having to consult the text.
