# Supplementary material for: Genomic surveillance of antimicrobial resistance shows cattle and poultry are a moderate source of multi-drug resistant non-typhoidal Salmonella in Mexico
Source: PLoS One. 2021 May 5;16(5):e0243681. doi: 10.1371/journal.pone.0243681 (PMC8099073; doi:10.1371/journal.pone.0243681)
Supplement: Attachment — Submitted filename: Response to reviewers R3.docx [file pone.0243681.s010.docx]

PONE-D-20-36934R2

Genomic surveillance of antimicrobial resistance shows cattle and poultry are a moderate source of multi-drug resistant non-typhoidal Salmonella in Mexico

PLOS ONE

The authors thank the academic editor and the reviewer for their detailed review. Below we list each comment raised during the reviewing process, followed by the authors’ responses**.**

**Reviewer Comments (RC)**

***RC1****. I believe the title of the manuscript needs a small revision (beside cattle, addressing the poultry as well as one of the sources based on their edits on data and results). At the discussion, the authors also acknowledge this by stating: " Likewise, our comparative analysis showed NTS strains isolated from cattle and poultry have strong AMR genotypes, which are similar to that of human clinical isolates.". I think the manuscript is almost ready for publication..*

**AR1**. Accepted. The title was modified to include poultry. Please, refer to the revised version of the manuscript.
